# Evaluation of the Effect of Platelet-Rich Plasma on the Outcome of IVF Cycle in Patients with Poor Ovarian Response

**DOI:** 10.5935/1518-0557.20250185

**Published:** 2026

**Authors:** Zahra Usefi, Mohammad Javad Azadchehr, Javad Amini Mahabadi, Tayebeh Hashemi Arani

**Affiliations:** 1 Anatomical Sciences Research Center, Institute for Basic Sciences, Kashan University of Medical Sciences, Kashan, Iran; 2 Infectious Diseases Research Center, Kashan University of Medical Sciences, Kashan, Iran

**Keywords:** infertility, PRP, IVF

## Abstract

**Objective:**

Platelet-rich plasma (PRP) is currently utilized in several clinical fields, including infertility treatment. This study aimed to evaluate the effectiveness of intraovarian PRP injections in improving reproductive outcomes in women who have previously experienced unsuccessful IVF cycles.

**Methods:**

In this Open-label Single-Arm Longitudinal clinical trial study, a number of 15 infertile patients, POSEIDON groups 1 and 3 (poor ovarian reserve), with no history of hematological, immunological, hormonal disorders, chromosomal and genetic abnormalities, and kidney failure were investigated. PRP was prepared using a standardized protocol and administered at a dose of 2.5 cc per ovary. Key reproductive parameters, including follicle development, oocyte quality, embryo formation, and endometrial thickness were measured before PRP treatment, immediately after treatment, and three months post-treatment. After collecting and sorting, the data was analyzed using SPSS Ver.26 statistical software.

**Results:**

Nearly 27% of the participants achieved pregnancy, despite previous IVF failures. The findings revealed significant improvements in the median number of follicles larger than 17 millimeters, the number of oocytes, fertilizable oocytes, and endometrial thickness following PRP treatment (*p*<0.05). The number of embryos formed, as well as the frequency of A or B grade embryos, also increased significantly at three months post-PRP (*p*<0.001).

**Conclusions:**

Intraovarian PRP injections show potential as an effective treatment for improving reproductive outcomes in women with a history of IVF failures. However, further research with larger sample sizes, control groups, and extended follow-up periods is needed to validate these findings and establish PRP as a standard therapy in reproductive medicine.

## INTRODUCTION

Infertility affects approximately 15% of couples globally (Mahabadi *et al.*, 2016). It is defined as the inability to conceive after at least one year of regular, unprotected intercourse, with a prevalence of around 9% among women (Mahabadi *et al.*, 2020). Infertility imposes significant psychological, physical, and emotional stress on families, potentially disrupting the social structure of the family unit. In recent years, factors such as delayed marriage and lifestyle changes, including increased exposure to environmental toxins, have contributed to rising infertility rates (Hanson *et al.*, 2017).

While a range of treatment options is available, advanced therapeutic approaches are often required when conventional methods prove unsuccessful. Among these, in vitro fertilization (IVF) remains a cornerstone, with an estimated success rate of 30%. Although embryo culture and transfer techniques have been refined over the past two decades in IVF clinics, the anticipated improvement in clinical pregnancy rates has not materialized (de Mouzon *et al.*, 2010). Recurrent implantation failure (RIF) continues to pose a significant challenge for both clinicians and patients in fertility treatment centers. RIF is typically defined as the failure to achieve pregnancy after three IVF cycles or following the transfer of ten high-quality embryos (Coughlan *et al.*, 2014). The underlying causes may include uterine factors such as a thin endometrium, poor endometrial receptivity, immune incompatibilities, and issues related to embryonic development.

Various treatment strategies have been explored for managing a thin endometrium, with the most recent approach involving intrauterine platelet-rich plasma (PRP) injection (Stamenov *et al.*, 2017; Taheripanah *et al.*, 2017; Zadehmodarres *et al.*, 2017). When platelets within PRP are activated, they release cytokines and growth factors within 10 minutes of clotting, which can enhance cellular migration, adhesion, proliferation, and differentiation, as well as accelerate extracellular matrix formation (Lee *et al.*, 2013). PRP is currently utilized in several clinical fields, including orthopedics, ophthalmology, and wound healing (Dhillon *et al.*, 2012). Recently, its application has extended to infertility treatment (Coksuer *et al.*, 2019; Sfakianoudis *et al.*, 2019; Nazari *et al.*, 2020). Numerous studies have investigated the effects of intrauterine PRP injection on pregnancy outcomes. Although limited, animal studies have demonstrated beneficial effects of intrauterine PRP injection on the endometrium and embryo, showing improved pregnancy rates and reduced endometrial inflammation (Marini *et al.*, 2016; Reghini *et al.*, 2016). In human studies, intrauterine PRP injection performed 48 hours before frozen embryo transfer in 20 and 97 patients with recurrent implantation failure resulted in pregnancy rates of approximately 80% and 44.89%, respectively (Nazari *et al.*, 2016, 2020). Other studies have reported that intrauterine PRP injection in 5 and 10 patients with inadequate endometrial growth during hormone therapy in embryo transfer cycles led to significant endometrial thickening and improved pregnancy outcomes in all participants (Zadehmodarres *et al.*, 2017). A previous meta-analysis reported a significant benefit in terms of increased pregnancy rates and endometrial thickness (Maleki-Hajiagha *et al.*, 2020).

While considerable research has focused on the effects of intrauterine PRP injections on pregnancy outcomes, there has been limited exploration of intraovarian PRP injections. The potential of PRP to improve pregnancy outcomes may lie in its ability to enhance the morphological quality of oocytes and embryos following intraovarian injection. This potential is largely attributed to the diverse range of growth factors and cytokines present in PRP, including interleukin-8, platelet-derived angiogenic factor, PDGF, IGF, stromal cell-derived factor-1 (SDF-1), vascular endothelial growth factor, fibroblast growth factor, epidermal growth factor, transforming growth factor-beta (TGF-β), fibronectin, vitronectin, and sphingosine-1-phosphate. These molecules are involved in key processes such as chemotaxis, controlled release of growth factors, angiogenesis, mitogenesis, and extracellular matrix formation (Urman *et al.*, 2019; Everts *et al.*, 2020), all of which may contribute to improved pregnancy outcomes. This potential has been highlighted in a recent study (Barrenetxea *et al.*, 2024), which has shifted focus towards intraovarian PRP injections, emphasizing the need for further clinical studies in this area.

Recently, intraovarian PRP injection has emerged as a novel approach aiming to rejuvenate ovarian function in patients with diminished ovarian reserve. Unlike intrauterine PRP, which primarily targets endometrial receptivity, intraovarian PRP is hypothesized to promote folliculogenesis through its rich content of growth factors and cytokines. However, clinical evidence remains limited, highlighting the need for studies such as ours to explore its efficacy in poor responders undergoing IVF (Navali *et al.*, 2023; Sadeghpour *et al.*, 2025).

This study focuses on patients with poor ovarian reserve, defined by AMH levels less than 1.2 ng/ml and AFC less than 5 (Poseidon Group 3), or a history of a previous poor response to ovarian stimulation. According to the Poseidon criteria, this group is categorized as having a poor ovarian reserve and is identified as requiring more specific interventions. Poseidon Group 3 specifically includes patients who face greater challenges with ovarian response and are more likely to have poor outcomes with conventional IVF protocols. The aim of this study is to investigate the effectiveness of intrauterine PRP injection as a novel treatment to improve IVF outcomes in these patients.

Given the scarcity of studies on infertility related to recurrent implantation failure and the promising success rates, efficacy, and safety of PRP injections, primarily due to their autologous origin, this study aims to assess the effectiveness of PRP and its impact on IVF cycle outcomes in poor responders.

## MATERIALS AND METHODS

### Trial Design

This study was conducted as an Open-label Single-Arm Longitudinal clinical trial, designed to evaluate of the effect of PRP on the outcome of IVF cycle in poorly responding patients.

### Blinding

In this study, neither the patients nor the physicians were blinded to the treatment. Both parties were fully informed about the procedures being performed and the specific interventions being administered. This open-label design was chosen due to the nature of the intervention, which required active participation and informed consent from the patients.

### Participants

The study included 15 patients aged under 35 years with BMI less than 30, Previous poor response and Poor ovarian reserve (AMH<1.2 ng/ml, and AFC<5 (Poseidon Group 3) or history of one previous poor response during an IVF cycle despite having AMH>1.2 ng/ml and AFC>5 (Poseidon Group 1) recruited from the infertility clinic of Shahid Beheshti Hospital, affiliated with Kashan University of Medical Sciences. Patients were excluded with Hematologic disorders (leukemia, thrombocytopenia), immunologic disorders (antiphospholipid syndrome, thrombophilia), hormonal disorders (diabetes, thyroid disorders, hyperprolactinemia), chromosomal and genetic abnormalities (hereditary or congenital), and renal failure.

### PRP Preparation Protocol

To prepare the PRP, 35 mL of venous blood was drawn from each patient using a sterile technique. The blood was collected directly into the PRP kit provided by ROOYAGEN fertilize lympho-PRP kit.

The PRP preparation involved a two-step centrifugation process as outlined by the kit instructions:

1. **First Spin:** The initial centrifugation was conducted at ∼280×g for 10 minutes to separate the plasma from the cellular components.
2. **Second Spin:** The plasma layer was then transferred to a new compartment within the kit and subjected to a second centrifugation at ∼1350×g for 6 minutes to concentrate the platelets.

After the second centrifugation, the PRP layer was carefully extracted following the kit’s guidelines. A total volume of 2.5 mL PRP was prepared for each patient. The prepared PRP was immediately used for injection into the ovaries.

The entire process was performed under sterile conditions, strictly adhering to the manufacturer’s protocol to ensure the integrity and efficacy of the PRP.

### Interventions

After obtaining informed consent and before entering the IVF cycle, patients underwent hormonal tests for FSH and AMH. The IVF cycle began with the administration of HMG at a dose of 300 IU/day, and a follow-up ultrasound was performed on the fifth day of stimulation. If a follicle measuring 12 to 14 millimeters was observed, the patient was prescribed 0.25 mg of Cetrotide. When two or more follicles measuring 17 millimeters or more were detected on ultrasound, final follicular stimulation was induced with the administration of 10,000 IU of HCG plus 0.2 mg of Decapeptyl. Thirty-six hours after final follicular stimulation, ovarian puncture was performed. Following the puncture and oocyte freezing, 2.5 cc of PRP was injected into each ovary. After PRP injection, an antagonist IVF cycle was conducted, and embryo transfer was performed.

### Outcomes

The primary outcome was occurrence of pregnancy. Secondary outcomes included the number of follicles greater than 17 mm, oocytes, fertile oocytes, embryos, Embryo quality (A&B), endometrial thickness. Before PRP, the first cycle after PRP, and three months after PRP, outcomes were measured.

### Statistical Methods

Descriptive statistics were used to summarize the data. Quantitative (continuous) variables were expressed as means±standard deviations, while qualitative (categorical) variables were described using frequencies (percentages). The Shapiro-Wilk test was employed to assess the normality of the distribution of the endometrial thickness variable across three different time points. Due to the non-normal distribution of this variable, non-parametric tests were utilized for further analysis.

For the comparison of outcome variables across the three time points, the following statistical tests were applied:

- Friedman test was used to assess changes over time for continuous variables with non-normal distribution.
- Repeated Poisson regression via Generalized Estimating Equations (GEE) was employed for count data such as the number of follicles, oocytes, and embryos.
- Repeated Logistic regression via GEE was applied for binary outcome variables, such as the quality of embryos (A&B).

Post-hoc pairwise comparisons were conducted using the Bonferroni correction to adjust for multiple testing. The results of these comparisons are reported as p-values for each pair of time points. The frequency of pregnancy occurrence was calculated and reported as a percentage of the total number of patients. A significance level of 0.05 was used for all statistical tests. All analyses were performed using SPSS version 26.0.

## RESULTS

This study was conducted with the aim of evaluation of the effect of PRP on the outcome of IVF cycle in poorly responding patients. Fifteen participants after matching the inclusion and exclusion criteria, entered the study.

Table 1 shows the characteristics and infertility information of participants. The mean age of the women in the study was 30.40±2.72 years, and their mean BMI was 27.58±4.06 kg/m^2^. Additionally, the mean duration of infertility among the participants was 3.93±1.22 years. Sixty percent of the women had primary infertility, with the most common cause of infertility being decreased ovarian reserve. Furthermore, the majority of women had experienced between 2 to 4 failed attempts at IUI or IVF. All participants had at least one prior IVF failure (Table 1 shows 2-8 failed attempts). Three of the four pregnancies resulted in live births, while one ended in a first-trimester miscarriage. The semen analysis in the four cases who achieved pregnancy, were normal. In all four pregnancies, semen analysis was normal (as per WHO 2010 criteria).

**Table 1:** Descriptive Characteristics of the Studied Patients.

Variable		Frequency (Percentage)/Mean±Standard Deviation (MIN-MAX)
**Age (years)**		30.40±2.72 (24-34)
**BMI (kg/m^2^)**		27.58±4.06 (19-36)
**Duration of Infertility (years)**		3.93±1.22 (2-6)
**Type of Infertility**	Primary	9 (60)
	Secondary	6 (40)
**Cause of Infertility**	Decreased Ovarian Reserve	9 (60)
	Decreased Ovarian Reserve+Partner Issues	3 (33.3)
	Endometrioma	1 (6.7)
**Semen Analysis**	Normal	11 (73.3)
	Abnormal	4 (26.7)
**Number of Failed IUI/IVF Attempts**	2	4 (26.7)
	3	4 (26.7)
	4	5 (33.3)
	5	1 (6.7)
	8	1 (6.7)

The Shapiro-Wilk test was used to assess the normality of the endometrial thickness variable at three different time points. The *p*-value at all three time points is less than the significance level of 0.05, suggesting that the distribution of the endometrial thickness variable is non-normal (Table 2).

**Table 2:** Shapiro-Wilk Test Results for Endometrial Thickness.

Time Point	Shapiro-Wilk Statistic (p-value)
**Before PRP**	0.869 (0.033)
**After PRP**	0.840 (0.013)
**Three months after PRP**	0.877 (0.043)

Table 3 shows the median number of follicles larger than 17 millimeters showed a significant increase in the second and third time points compared to the first time point (*p*<0.05). Additionally, the median number of oocytes, fertilizable oocytes, and endometrial thickness significantly increased in each pair of time points (*p*<0.05). The median number of embryos also significantly increased in the third time point compared to the first and second time points (*p*<0.05). Furthermore, the frequency of A or B grade embryos significantly increased in the third time point compared to the first and second time points (*p*<0.05). Endometrial thickness also showed a significant increase in the second and third time points compared to the first (*p*<0.011).

**Table 3:** Comparison of Secondary outcomes Across Three Different Time Points.

Variable	Before PRP	Cycle on PRP	Three months after PRP	p-value	Pairwise Comparisons
**Number of Follicles>17 mm**	2 (2-3)	3 (2-5)	3 (3-4)	0.070^*^	1st-2nd: *p*=0.023 1st-3rd: *p*=0.040 2nd-3rd: *p*=0.869
**Number of Oocytes**	1 (0-2)	2 (1-2)	2 (2-4)	<0.001^*^	1st-2nd: *p*=0.008 1st-3rd: *p*<0.001 2nd-3rd: *p*=0.003
**Number of Fertilizable Oocytes**	0 (0-1)	0 (0-2)	2 (1-3)	<0.001^*^	1st-2nd: *p*=0.022 1st-3rd: *p*<0.001 2nd-3rd: *p*=0.010
**Number of Embryos**	0 (0-1)	0 (0-1)	2 (1-3)	<0.001^*^	1st-2nd: *p*=0.125 1st-3rd: *p*<0.001 2nd-3rd: *p*=0.009
**Quality of Embryos (A&B)**	3 (20%)	5 (33.3%)	13 (86.7%)	0.001^**^	1st-2nd: *p*=0.310 1st-3rd: *p*<0.001 2nd-3rd: *p*=0.001
**Endometrial Thickness**	7 (7-8)	8 (8-10)	12 (10-14)	<0.001^***^	1st-2nd: *p*=0.011 1st-3rd: *p*<0.001 2nd-3rd: *p*<0.001
•The data in the table are reported as frequency (percentage) or median (interquartile range). •The first, second, and third time points refer to before PRP, First cycle after PRP, and three months after PRP, respectively. •Repeated Poisson regression via GEE/^**^ Repeated logistic regression via GEE/^***^ Friedman test					

Table 4 presents the primary outcomes of the study. Approximately 27% of the women in the study became pregnant.

**Table 4:** Frequency of Pregnancy Occurrence in the Studied Patients.

Variable	Frequency (%)
**Pregnancy**	4 (26.7)

## DISCUSSION

Infertility imposes significant psychological, physical, and emotional burdens on families and can have detrimental effects on the social fabric. In recent years, factors such as delayed marriage and lifestyle changes, including increased exposure to environmental toxins, have contributed to the rising rates of infertility (Hanson *et al.*, 2017). The PRP technique is one of the latest methods shown to be effective in treating infertility, with promising results observed in intrauterine application ns (Stamenov *et al.*, 2017; Taheripanah *et al.*, 2017; Zadehmodarres *et al.*, 2017; Sfakianoudis *et al.*, 2019; Nazari *et al.*, 2020). PRP works by activating platelets, which then release cytokines and growth factors within 10 minutes of clot formation. These bioactive molecules can regulate processes such as cell migration, adhesion, proliferation, and differentiation, while also accelerating the formation of the extracellular matrix (Lee *et al.*, 2013). Recent studies have proposed that PRP injections directly into the ovaries after ovarian puncture could enhance hormonal and embryonic parameters. Therefore, this study focused on intraovarian PRP injections, acknowledging the need for further research in this area.

The primary objective of this study was to evaluate the effectiveness of intraovarian PRP injections in improving pregnancy outcomes in women who had previously experienced IVF treatment failures. The findings of this study showed that nearly 27% of the women, most of whom had experienced 2 to 4 IVF treatment failures, became pregnant following PRP treatment. This is a notable improvement compared to the study by Shahrokh Tehraninejad *et al.* (2023), where only 3% of patients in the PRP group achieved pregnancy. The disparity in results may be attributed to differences in the study populations, as patients in Shahrokh Tehraninejad *et al.* (2023) study were generally older and had a lower ovarian reserve, factors that are known to negatively impact fertility outcomes. Similarly, in the study by Tülek & Kahraman (2022), the clinical pregnancy rate was reported to be 8.3% after PRP treatment. The variation in outcomes between these studies could also be due to differences in the PRP administration technique; in our study, 2.5 cc of PRP was injected into each ovary, whereas Tülek & Kahraman (2022) study used a smaller volume of 2 cc per ovary. These findings suggest that the volume of PRP and the specific characteristics of the patient population may play critical roles in the success of PRP treatment for enhancing fertility. Given these results, further research is warranted to optimize PRP protocols and to better understand the patient factors that contribute to successful outcomes. The disparity in results may be attributed to differences in the study populations, as patients in Shahrokh Tehraninejad *et al.* (2023) study were generally older and had a lower ovarian reserve, factors known to negatively impact fertility outcomes. Similarly, in the study by Tülek & Kahraman (2022), the clinical pregnancy rate was reported to be 8.3% after PRP treatment. Although the difference in PRP volume between our study (2.5 cc per ovary) and Tülek & Kahraman (2022) study (2 cc per ovary) might have influenced the results, we recognize that other factors such as patient age, AMH levels, sample size, and baseline ovarian function likely played substantial roles. In both of those studies, the included patients were generally older and had more severely diminished ovarian reserve compared to our cohort. These demographic and biological differences could account for the lower pregnancy rates observed in those studies, regardless of the PRP volume or protocol used.

The study observed a significant increase in the median number of follicles larger than 17 millimeters at the second and third time points compared to the first. Additionally, the number of oocytes and fertilizable oocytes increased significantly following PRP injection. This suggests that injecting 2.5 cc of PRP into each ovary enhances the number of follicles suitable for pregnancy in subsequent cycles, lasting up to three months after the injection. Shrivastava *et al.* (2024) found similar results, where PRP treatment led to an increase in follicle numbers, improved oocyte quality, and successful pregnancy outcomes in the subsequent IVF cycle. PRP treatment has shown promise in fertility therapies, particularly in increasing ovarian reserve, improving oocyte quality, and enhancing successful pregnancy outcomes. Similarly, Navali *et al.* (2023), in a clinical trial involving 35 patients with poor ovarian response (POR), reported findings consistent with our study, showing an improvement in the number of fertilizable oocytes after PRP injection. Farimani *et al.* (2019), in a non-randomized clinical trial involving 56 women with a history of POR, reported an increase in the mean number of oocytes from 0.64 to 2.1 before and after PRP injection. In a study by Cakiroglu *et al.* (2022) involving 510 women aged 30 to 45 years with decreased ovarian reserve, the number of mature oocytes increased from 1.7 to 2.7 following PRP treatment. However, Shahrokh Tehraninejad *et al.* (2023) found no significant difference in oocyte quality between the PRP intervention group and the control group. The discrepancy between Shahrokh Tehraninejad *et al.* (2023) study and ours could be attributed to differences in the study populations; Shahrokh Tehraninejad *et al.* (2023) study involved older patients with lower ovarian reserve (lower AMH levels), which likely influenced the outcomes. It appears that age and initial ovarian reserve are two critical factors affecting the quality of follicles suitable for pregnancy. As known, PRP is essentially a concentrate of the patient’s blood with a higher concentration of platelets. Additionally, PRP contains several other components that play significant roles in its mechanism of action. Platelet-derived growth factor (PDGF), for instance, is crucial as it is one of the first components to be present at the site of tissue injury. PDGF is known for its role in enhancing cellular recruitment, proliferation, and differentiation, while also contributing to angiogenesis and inflammation (Du *et al.*, 2018). Transforming growth factor (TGF), on the other hand, plays a role in initiating tissue regeneration and acts as a stimulant for tissue repair (Gonçalves *et al.*, 2016). Moreover, TGF can activate vascular endothelial growth factor (VEGF), which is instrumental in angiogenesis (Zhang *et al.*, 2016). Given the current understanding, the combination of ovarian angiogenesis and the pivotal role of PDGF in activating and stabilizing blood vessels suggests that autologous PRP therapy may facilitate ovarian tissue regeneration. This could be the underlying reason for the improvement in the number of follicles larger than 17 millimeters, as well as the increase in oocytes, particularly those suitable for fertilization.

In this study, embryonic outcomes were also assessed. The median number of embryos significantly increased at the third time point compared to the first and second. This suggests that three months after PRP treatment, the number of embryos formed was significantly higher than before PRP and in the immediate post-PRP cycle. Additionally, the frequency of A or B grade embryos significantly increased at the third time point compared to the earlier time points, indicating an improvement in embryo quality following PRP treatment. These findings align with previous studies. For example, Cakiroglu *et al.* (2022) reported an increase in the number of embryos formed and an improvement in embryo growth quality. A meta-analysis by Li *et al.* (2023), which reviewed 10 studies involving 793 patients with decreased ovarian reserve, also found an increase in the number of embryos formed two months after PRP, consistent with our study. Similarly, Tülek & Kahraman (2022), who evaluated 71 patients with decreased ovarian reserve, reported a higher number of pronuclear and cleavage stage embryos after PRP, which supports our findings. Parvanov *et al.* (2022) also reported better-quality embryos formed after PRP in a study involving 66 patients with POR. The mechanism by which PRP improves oocyte and embryo morphology following intraovarian injection likely involves the broad activity of the growth factors and cytokines present in PRP. These include interleukin-8, platelet-derived angiogenic factor, PDGF, IGF, stromal cell-derived factor-1 (SDF-1), VEGF, fibroblast growth factor, epidermal growth factor, transforming growth factor-beta (TGF-β), fibronectin, vitronectin, and sphingosine-1-phosphate (Dawood & Salem, 2018; Urman *et al.*, 2019). These molecules contribute to improved embryo quality through processes such as chemotaxis, controlled release of various growth factors, angiogenesis, mitogenesis, and extracellular matrix formation (Everts *et al.*, 2020). For instance, SDF-1 supports the recruitment of CD-34 positive precursor cells and tissue regeneration (Stellos *et al.*, 2008), while PDGF plays a critical role in directing cellular migration (Yamada *et al.*, 2018). Additionally, PDGF is involved in overcoming growth arrest by promoting DNA synthesis and mitosis (Zhao *et al.*, 2016). It has been proposed that these specific components of PRP may provide the necessary signals to induce the differentiation of progenitor or stem cells into mature oocytes (Sills & Wood, 2019).

This study also evaluated endometrial thickness, which showed a significant increase at the second and third time points compared to the first. This indicates that endometrial thickness increased after PRP injection, with further growth observed over the subsequent three months. In a previous study by Huniadi *et al.* (2023) on 51 patients with endometrial thickness less than 7 millimeters, the results showed an increase in endometrial thickness after PRP injection in uterin cavity. Eftekhar *et al.* (2018) also reported an increase in endometrial thickness following PRP injection in uterine, along with significantly higher implantation and clinical pregnancy rates. Maleki-Hajiagha *et al.* (2020), in a meta-analysis, examined the effects of intrauterine PRP injection on pregnancy outcomes and endometrial thickness in women with recurrent implantation failure, reporting a significant positive effect of PRP in increasing both pregnancy rates and endometrial thickness. Several mechanisms have been proposed to explain the increase in endometrial thickness observed after PRP, including a) activation of dormant follicles, b) increased cortical volume, and c) induction of neoangiogenesis in dysfunctional ovarian tissue. All these factors enhance ovarian hormonal function, contributing to increased endometrial thickness.

Overall, the results of this study suggest that intraovarian PRP injections have a promising role in enhancing ovarian function, improving oocyte quality, and increasing the likelihood of successful pregnancy in women who have previously experienced IVF failures. The significant improvements observed in follicle development, oocyte yield, embryo quality, and endometrial thickness highlight the potential of PRP as an adjunctive treatment in assisted reproductive technologies. However, despite these encouraging findings, it is important to acknowledge the need for larger, randomized controlled trials to validate these outcomes and to optimize the PRP treatment protocols. Understanding the specific patient characteristics that predict better responses to PRP will also be crucial in tailoring individualized fertility treatments. As the field of reproductive medicine continues to evolve, PRP therapy could emerge as a key tool in overcoming the challenges associated with poor ovarian response and recurrent implantation failure, offering renewed hope to patients struggling with infertility.

Despite the promising findings of this study, several limitations must be acknowledged. First, the sample size was relatively small, which may affect the generalizability of the results to a larger population. Additionally, this research was conducted at a single center, potentially introducing selection bias and limiting the applicability of the findings across different settings and populations. The follow-up period was limited to Three months, which restricts the ability to evaluate the long-term effects of PRP injections on ovarian function and pregnancy outcomes. Also, platelet concentration was not routinely measured post-PRP due to kit limitations (ROOYAGEN provides standardized PRP). Furthermore, there may have been variability in the preparation and administration of PRP, even though a standardized protocol was followed, potentially influencing the consistency of the results. Lastly, the heterogeneity of the patient population, including variations in age and ovarian reserve, could have impacted the outcomes. Future studies with larger, randomized controlled trials, multi-center collaboration, extended follow-up durations, and more homogeneous patient groups are necessary to validate and expand upon these findings.

## CONCLUSION

This study provides valuable insights into the potential benefits of intraovarian PRP injections for women who have experienced previous IVF treatment failures. The findings demonstrate that PRP treatment significantly improves several key reproductive parameters, including follicle development, oocyte quality, embryo formation, and endometrial thickness. Nearly 27% of the women in this study, most of whom had undergone multiple unsuccessful IVF attempts, achieved pregnancy following PRP treatment. This suggests that PRP may play a crucial role in enhancing fertility outcomes, particularly in women with diminished ovarian reserve or poor ovarian response.

The observed improvements in reproductive outcomes can be attributed to the biological effects of PRP, which includes the activation of growth factors and cytokines that promote cellular proliferation, tissue regeneration, and angiogenesis. These mechanisms likely contribute to the enhanced ovarian function and endometrial environment observed in this study.

Despite these promising results, the study also highlights the need for further research to optimize PRP protocols and to better understand the patient-specific factors that influence treatment success. Larger, controlled trials with longer follow-up periods are essential to validate these findings and to establish PRP as a standard treatment option in reproductive medicine.

In conclusion, intraovarian PRP injections represent a promising adjunctive therapy for improving pregnancy outcomes in women with a history of IVF failures. As reproductive technologies continue to evolve, PRP may offer new hope to patients facing the challenges of infertility, potentially expanding the range of effective treatments available in clinical practice.

## Abbreviations

IVF: In Vitro Fertilization;

RIF: Recurrent Implantation Failure;

PRP: Platelet-Rich Plasma;

GEE: Generalized Estimating Equations;

PDGF: Platelet-Derived Growth Factor;

TGF: Transforming Growth Factor;

VEGF: Vascular Endothelial Growth Factor;

SDF-1: Stromal Cell-Derived Factor-1;

TGF-β: Transforming Growth Factor-Beta

AMH: Anti-Müllerian Hormone;

AFC: Antral Follicle Count;
